# Establishment and characterization of patient-derived tongue squamous cell carcinoma cell lines

**DOI:** 10.1007/s13577-025-01231-w

**Published:** 2025-05-20

**Authors:** Priyanka Joshi, Sanjay Bane, Pankaj Chaturvedi, Poonam Gera, Sanjeev K. Waghmare

**Affiliations:** 1 Stem Cell Biology Group, Waghmare Lab, Advanced Centre for Treatment Research and Education in Cancer (ACTREC), Cancer Research Institute, Tata Memorial Centre, Kharghar, Navi Mumbai, 410210 Maharashtra India; 2 Homi Bhabha National Institute, Training School Complex, Anushakti Nagar, Mumbai, 400085 India; 3 Biorepository, Advanced Centre for Treatment, Research and Education in Cancer, Tata Memorial Centre, Navi Mumbai, 410210 India; 4 Advanced Centre for Treatment Research and Education in Cancer (ACTREC), Tata Memorial Centre, Kharghar, Navi Mumbai, 410210 Maharashtra India; 5 Tata Memorial Hospital, Homi Bhabha National Institute, Mumbai, India

**Keywords:** Tongue carcinoma, Cell line, Epithelial–mesenchymal transition, Cancer stem cells, In vivo tumorigenesis

## Abstract

Oral squamous cell carcinoma (OSCC) is a common carcinoma in Indian population, wherein one-third of global OSCC cases are from India. The five-year survival rate is poor due to late diagnosis. Oral tongue squamous cell carcinoma (OTSCC) is the second-most common OSCC. An in vitro cell line model is a valuable tool to get a deeper understanding of the molecular mechanisms involved in therapy resistance and disease progression. We report establishment of three OTSCC cell lines from advanced stage treatment naïve Indian patient samples, such as ACOTSC120, ACOSTC132, and ACOTSC140. All three OTSCC cell lines showed epithelial morphology, which was confirmed by Keratin-14 staining. The cell lines showed in vitro spheroid-forming and in vivo tumorigenic potential. The STR of the cell lines ensured their human origin and novelty when compared to DSMZ cell line database. The karyotype of the cell lines showed aneuploidy and further confirmed their human origin. These cell lines showed the presence of cancer stem cell (CSCs) population, i.e., the ALDH^br^/CD44^+^ population. These cell lines thus provide a model to help understand the biology of disease and its progression.

**Supplementary Information:**

The online version contains supplementary material available at 10.1007/s13577-025-01231-w.

## Introduction

Oral squamous cell carcinoma (OSCC) is the sixth most common cancer in the world [1], wherein one-third cases come from India, making it a serious health risk in India. A high prevalence is due to lack of awareness regarding risk factors and unavailability of early detection services [2]. Around 70% of the cases are diagnosed in the later stage bringing down the five-year survival to around 20% [1]. Among OSCC, oral tongue squamous cell carcinoma (OTSCC) is second most common. Currently employed treatment regime against OSCC consists of surgery, chemo-radiotherapy, and targeted therapy. Surgery is considered as a primary treatment modality in majority of cases and performed in combination with chemo-radiation to improve treatment outcome [3]. Surgery is preferred in cases of early stage disease, while complex procedures involving reconstruction and adjuvant chemo-radiotherapy are performed in cases of advanced stage cancers [4].

Although multi-modality treatments are utilized, the five-year survival rate has been poor for OSCC. An in vitro patient-derived OSCC cell line model is a promising tool that can help get a deeper understanding of this problem.

Previously, there have been reports of squamous cell carcinoma (SCC) cell lines established from patient OTSCC tissues. Patient-derived SCC cell lines have been established through explant culture [5]. Four out of six cell lines reported originated from OTSCC wherein three cell lines generated tumors in vivo in nude mice, while one formed cyst [5]. Furthermore, drug-resistant cell lines were established from two treatment naïve tongue carcinoma patient samples (Cal27 and Cal33) [6]. Two cell lines expressing major histocompatibility complex class I (MHC I) molecule were established from poorly differentiated tongue carcinoma (AW13516) and epidermoid tongue carcinoma (AW8507) [7]. In addition, another cell line was established from a Chinese OTSCC patient sample [8]. Two OTSCC cell lines were established from Japanese patients with advanced stage disease [9]. Tongue squamous cell carcinoma (TSCC) cell lines expressing epidermal growth factor receptor (EGFR) [10–12] as well as p53 mutation have been reported [12]. A radiosensitive cell line established from an OTSCC patient sample has also been reported [13]. Cell lines have also been established from the metastatic lymph nodes of OTSCC [14, 15]. Recently, cell lines have been established from patient sample (NOKT-1) and from the tumor generated in vivo(NOKT-1-XG) [16]. Previously, there have been Indian OTSCC cell lines reported, although, their in vivo tumorigenic potential was not demonstrated [17, 18].

In the present study, we report establishment and characterization of three cell lines (ACOTSC120, ACOTSC132, and ACOTSC140) from advanced stage treatment naïve OTSCC Indian patient samples. These cell lines may prove useful in understanding the biology of disease, which may aid in improving treatment outcome.

## Materials and methods

### Patient tissue collection and processing

The tissue samples of advanced stage, treatment naïve OTSCC patients were collected post-surgery after obtaining their informed consent. The exclusion criteria were to exclude human immuno-deficiency virus (HIV)-positive, hepatitis B virus (HBV)-positive infection, early-stage OTSCC, recurrent disease and prior cancer treatment. All procedures were approved by institutional ethics committee (IEC) under the project no. 900188. The clinico-pathological characteristics of patients are detailed in Supplementary Table 1. The tissue samples were surface-sterilized by 10% povidone-iodine (Wokadine™) and then washed with sterile 1 × phosphate buffered saline (PBS) followed by explant culture.

### Characterization of patient tumor

The patient tumors were fixed using 4% PFA and embedded in paraffin. Further, sections of 5 µm were cut to perform H&E staining and immunohistochemistry (IHC) for cytokeratin 14, keratin 8, E-cadherin, and vimentin. The Vectastain® Elite® ABC-HRP Kit (Peroxidase, Universal) (Vector laboratories, USA, PK-6200) was used to detect the antibody binding while 3,3-diaminobenzidine was used as substrate. The tissues were counterstained using hematoxylin and mounted using DPX mountant. The primary antibody concentration is detailed in Supplementary Table 5.

### Explant culture and cell culture

Following disinfection, the tissues were cut into smaller pieces, approximately 1 mm × 1 mm in dimension and placed in 35 mm tissue culture plates containing tissue culture medium (Minimal essential medium supplemented with 5% Hyclone FBS Cat. No. SH30071.03 and 5% Gibco horse serum Cat. No. 26050–088, USA). The explants were maintained as described in Gawas et al. [19], the keratinocytes were isolated by differential trypsinization and expanded. Briefly, the cells were passaged when they reached 70–80% confluency using 0.25% trypsin–EDTA and stored in the form of freeze-downs in liquid nitrogen in a complete medium with 10% dimethyl sulfoxide (DMSO) (Sigma).

### Calculation of doubling time

The doubling time was calculated as described in Gawas et al. [19] Briefly, the cells were counted at 24 h intervals for 96 h and the doubling time was calculated using following formula:

$${\text{Doubling}}{\mkern 1mu} {\text{Time}} = \frac{{{\text{duration}} * {\mkern 1mu} \log {\mkern 1mu} (2)}}{{\log {\mkern 1mu} {\mkern 1mu} \left( {{\text{Final}}{\mkern 1mu} {\text{Concentration}}} \right) - \log {\mkern 1mu} {\mkern 1mu} \left( {{\text{Initial}}{\mkern 1mu} {\text{Concentration}}} \right)}}$$

### STR profiling

STR profiling was done to authenticate the cell lines and ensure their human origin as described previously in Gawas et al. [19] The STR profile of patient tissue from which the cell lines were derived was also performed in order to ensure the origin of cell line from corresponding patient tumors (OSC120, OSC132 and OSC140). In short, DNA from cell lines and patient tumor was isolated using DNeasy Blood & Tissue Kit (Qiagen). The short tandem repeat (STR) profile of cell lines was done for 16 loci to establish the human origin of the cell lines and ensure that the cell lines are unique.

### Mycoplasma detection

To check for mycoplasma contamination, mycoplasma testing was also performed using the Mycoplasma Detection kit-Quick test (Biotool, Spain, Cat. No. B39032). Briefly, 10 µl of positive control, unused sterile culture medium and 48 h spent medium from the cell lines were incubated with 40 µl of buffer A for five minutes at room temperature (RT) followed by incubation with buffer B for 4 min at RT and then stop solution was added. The results were recorded after 30 min.

### Karyotyping and ploidy analysis

Karyotyping analysis was performed by the modifications in the GTG banding method used for fibroblast cells. In all three cell lines, 20 metaphases were counted and analyzed. For all three cell lines, 8, 11 and 12 metaphases were karyotyped, respectively.

Ploidy analysis was performed as described in Gawas et al. [19] Briefly, DNA content of cells in G_1_ phase was compared with that of human peripheral blood mononuclear cells (PBMCs) (Sigma-Aldrich; Merck KGaA) and the ploidy of cells was determined on the basis of DNA indices.

### HPV genotyping

Isolation of DNA was done from all three cell lines using DNeasy Blood & Tissue kit (Qiagen). The DNA was then subjected to nested PCR, wherein the first PCR reaction was set up with the MY09/MY11 primers. The 450 bp long product of this reaction was then used as template DNA for primer reactions Gp5 +/Gp6 +, (Supp. Table 4) producing 150 bp product [20]. The DNA from HeLa cells was used as a positive control.

### Immunofluorescence(IF) staining

The cells were grown on a coverslip up to 60–70% confluency and fixed with 4% PFA. Further, permeabilization was done by 0.1% PBST (PBS with 0.1% Triton X-100). The blocking was done by incubating cells with blocking solution which was 5% normal goat serum (NGS) in PBS for one hour at RT. The cells were then incubated at 4^0^C overnight with primary antibody. Further, the cells were washed thrice with PBS prior to incubation with the secondary antibodies for one hour. The primary and the secondary antibodies used are listed in Supplementary Table 5. The cells were then subjected to Hoechst staining (1:500) for 10 min at RT followed by three washes of PBS. The cells were then mounted with Antifade and observed under confocal microscope.

### Fluorescence activated cell sorting (FACS) analysis

The cells were trypsinized to obtain a single cell suspension followed by staining with viability dye Zombie Aqua (1:500) (Biolegend, USA) to eliminate dead cells. Further, ALDERED assay (EMD Millipore, USA) was performed to assess ALDH activity in the cell lines. The cells were then washed with PBS and stained with APC-conjugated anti-CD44 antibody (Suppl. Table 5) (5 μl/10^6^ cells) for half an hour on ice. The cells were then washed with PBS followed by resuspending in 5% FBS. The cells were analyzed on BD biosciences FACS Aria system.

### Orosphere assay

The orosphere assay was performed as detailed in Gawas et al. [19] Briefly, 10,000 cells were grown in ultralow attachment condition in Mammocult medium (Stemcell technologies, Canada) and the spheroids formed were counted.

### In vivo tumorigenesis assay

The in vivo tumorigenesis assay was performed as described in Gawas et al. [19] to assess the tumor forming ability of the cell lines. In brief, 2 X 10^6^ cells of all three cell lines were subcutaneously injected in non-obese diabetic severe combined-immunodeficient (NOD-SCID) female mice and the tumors formed were measured periodically using vernier caliper. The mice were sacrificed before the tumors reached the humane limit of tumor volume. The tumors were collected and stained with H&E staining method to assess the tumor content. The tumor volume was plotted and analyzed using Graphpad Prism 8.0.2

### RNA isolation and cDNA preparation

RNA isolation and cDNA preparation were done as detailed in Navarange et al. [21] Briefly, RNA was isolated from all three cell lines using Trizol reagent and cDNA preparation was done using Primescript™ RT reagent kit (Takarabio). The cDNA was further used for real-time polymerase chain reaction (QRT-PCR).

### qRT-PCR

The TB Green® Premix Ex Taq™ II (Tli RNase H Plus) (Takara, USA) kit was used to perform SYBR-green-based QRT-PCR. The expressions of genes were assessed with respect to GAPDH as described in Navarange et al. [21] The sequence of primers used for reactions is mentioned in the supplementary figures. (Supp. Table 4).

### Cell migration assay

Cell migration assay was performed as detailed in Navarange et al. [21] In short, migration properties of all three cell lines through polycarbonate filter disk using complete medium as chemoattractant were assessed using the Boyden chamber plate and staining the migrated cells using crystal violet.

### Invasion assay

Invasion assays were performed as detailed in Navarange et al. [21] Briefly, invasion property of all three cell lines through the Matrigel layer was assessed using complete medium as chemoattractant using the Boyden chamber plate and staining the invading cells using crystal violet.

### In vivo metastatic potential

In order to assess the in vivo metastatic potential of the cell lines, 60,000 cells of the three cell lines (ACOTSC120 P30, ACOTSC132 P30, ACOTSC140 P32) were orthotopically injected in female NOD-SCID mice (aged 5–6 weeks). The tumor growth was observed and the mice were sacrificed upon 20% body weight reduction. The peripheral local lymph nodes (LNs) (superficial cervical lymph nodes) were collected to assess for LN metastasis and lungs were collected to assess for distant metastasis. LNs and lungs were fixed using 4% PFA and embedded in paraffin followed by H&E staining for assessment of metastasis.

### Electron microscopy (EM)

All three cell lines were grown up to 90–100% confluency and fixed using 3% glutaraldehyde in 0.1 M sodium cacodylate–HCL, pH 7.4 for 2 h at 4^0^C followed by 1% osmium tetroxide for 1 h at 40 °C (both from Ted Pella, Inc, USA). The cells were dehydrated and stained with 2% aqueous uranyl acetate and assessed using transmission electron microscopy as described in Navarange et al. [21]

### Statistical analysis

The data generated from QRT-PCR, in vitro orosphere assay (number of orospheres/well), in vivo tumorigenesis (tumor volume), invasion assay, migration assay, EM and FACS analysis were statistically analyzed using Graphpad prism 8.0.2 (263) and mean ± SEM was plotted in graph. The data were analyzed using Student’s t test and the p values were represented as ^∗^:*p* < 0.05, ^∗∗^:*p* < 0.01, ^∗∗∗^:*p* < 0.001.

## Results

### Establishment and characterization of patient-derived OTSCC cell lines

**Fig. 1:**
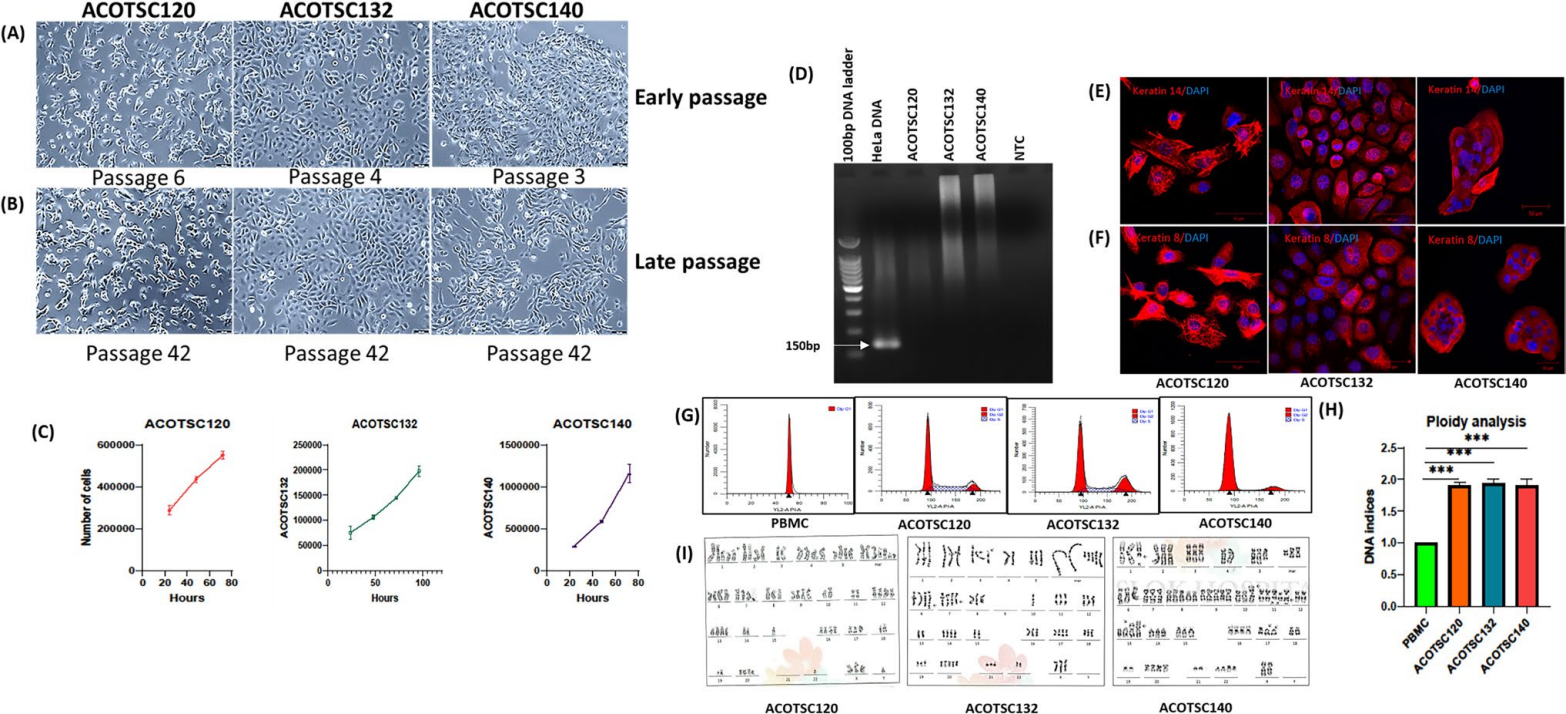
**A** Early passage of ACOTSC120 (P6), ACOTSC132 (P4) and ACOTSC140 (P3) and **B** late passage of ACOTSC120 (P42), ACOTSC132 (P42) and ACOTSC140 (P42) **C** the growth curve of the cell lines **D** The HPV genotype of the cell lines showing the 150 bp PCR product of GP5 +/GP6 + PCR **E** IF staining for cytokeratin 14 and **F** cytokeratin 8 for ACOTSC120, ACOTSC132 and ACOTSC140. The staining was carried out on three independent replicates for all three cell lines (P4, P5, P6). Representation of **G** ploidy analysis (P7,P8,P9) **H** Graphical representation of Ploidy analysis and **I** karyotype of the cell lines (P8). Student’s t test was employed for statistical analysis of the data and p values are represented as ^∗^:*p* < 0.05, ^∗∗^:*p* < 0.01, ^∗∗∗^:*p* < 0.001

All three cell lines (ACOTSC120, ACOTSC132 and ACOTSC140) were established from advanced stage treatment naïve OTSCC patient samples with a history of tobacco consumption. The clinical features of the cell lines are shown in (Suppl. Table 1). The patient tumors were characterized by performing H&E staining and IHC staining for Keratin 14 and Keratin 8 (Suppl. Figure 1A). The cells were polygonal in shape. ACOTSC120 showed growth in loose clusters while ACOTSC132 and ACOTSC140 grew in more compact clusters (Fig. 1A). All three cell lines were passaged over 40 passages (Fig. 1B) and were immortal. The STR profile of all three cell lines and corresponding patient samples (OSC120, OSC132 and OSC140) was unique than any prior reported cell line in the DSMZ database or with each other (Suppl. Table 2). The STR profile of the cell lines and the corresponding patient samples were identical to each other. Although the STR profiling did not report any mycoplasma contamination, we also performed test using kit method to ensure that the cells were free of mycoplasma contamination (Suppl. Figure 2). The doubling time of ACOTSC120, ACOTSC132 and ACOTSC140 was calculated that was 82.34, 91.16 and 33.83 h, respectively (Fig. 1C). All three cell lines were checked for HPV by nested PCR using the standard MY09/MY11 and GP5 +/GP6 + primers that showed the cell lines were HPV-negative (Fig. 1D).

The epithelial nature of the cells was assessed by performing Keratin 14 IF staining which is a marker for stratified epithelium [22]. Expression of Keratin 8 was evaluated in all three cell lines as a marker for dysplastic and tumor cells [23]. All three cell lines exhibited strong and uniform expression of Keratin 14 (Fig. 1E). Expression of Keratin 8 was seen to be stronger and evenly distributed in ACOTSC120 while ACOTSC132 and ACOTSC140 showed comparatively uneven expression (Fig. 1F). Genome instability is a hallmark of cancer [24]. Therefore, karyotype and ploidy analysis of the cell lines was done. The ploidy analysis was done as detailed in Gawas et al. [19] All three cells showed hyperploidy as compared to PBMCs with DNA indices of 1.81,1.82, and 1.72 for ACOTSC120, ACOTSC132, and ACOTSC140, respectively (Fig. 1G, H). Karyotype analysis of all three lines displayed abnormal ploidy. ACOTSC120 showed hyperploidy with 65–83 chromosomes, deletion of 1p and 21 chromosome and presence of dicentric chromosomes and minutes. Both ACOTSC132 and ACOTSC140 showed near triploidy with 59–79 chromosomes and 63–80 chromosomes respectively and also presence of dicentric chromosomes. Other aberrations observed in ACOTSC132 include homogenously stained regions (HSRs), additional material on chromosomes (1p, 6q, 12q and 16q), deletions in 3p and multiple copies of chromosome 9. In the ACOTSC140 cells, loss of Y chromosome was seen along with deletions in 1p, 1q, or isochromosome 1q, 8p, t(10;13)(q26.3;p11), 17p(p53) (Fig. 1I).

### In vivo tumorigenesis

**Fig. 2:**
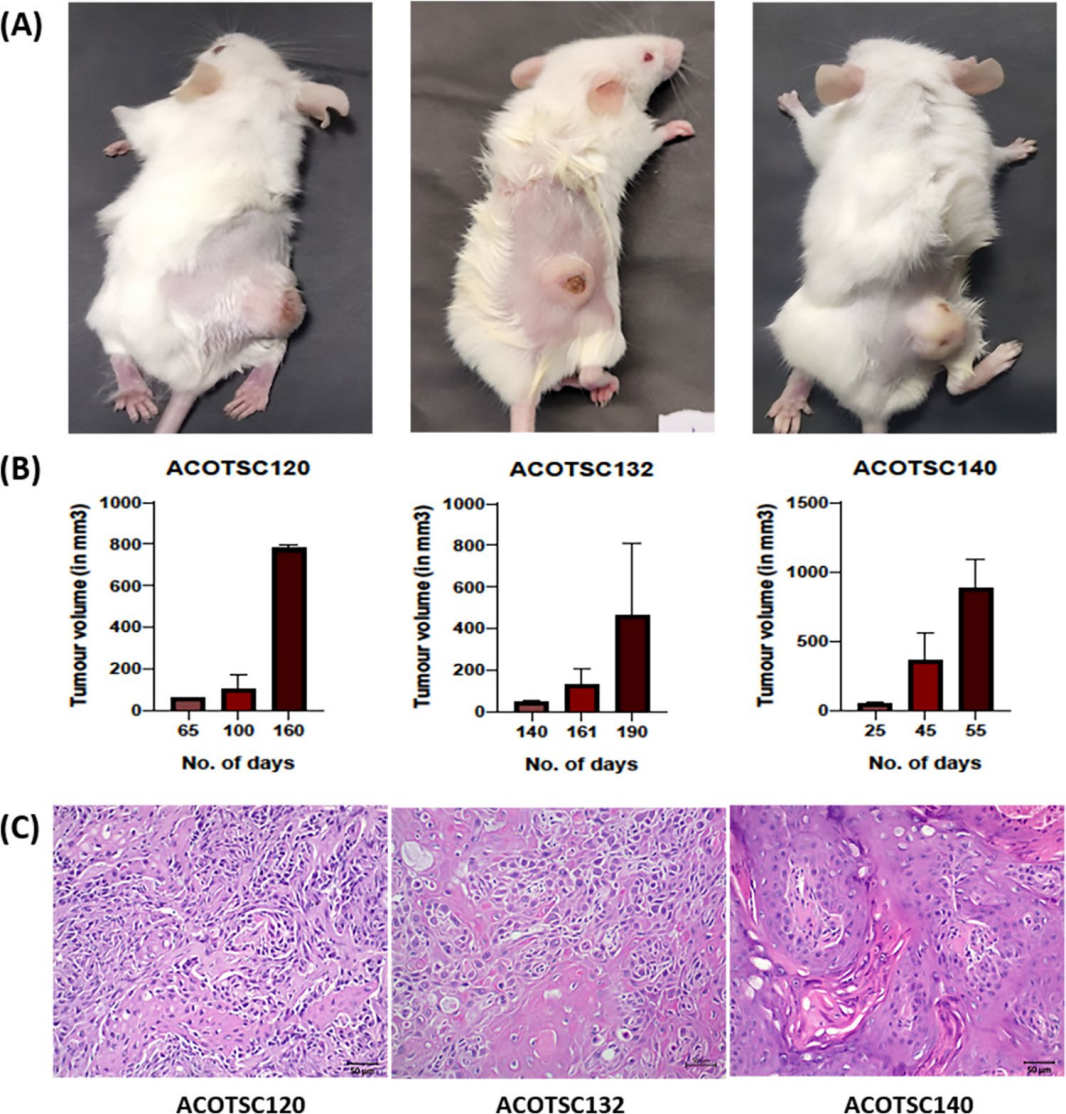
**A** The in vivo tumorigenesis assay of ACOTSC120 (P12), ACOTSC132 (P13) and ACOTSC140 (P14) cell lines injected in mice (*n* = 3 independent biological replicates for each cell line). **B** The tumor volume of the tumors generated in vivo. **C** The H&E staining of the tumors generated

To assess whether all the three cell lines have tumorigenic potential, we injected the three cell lines in NOD/SCID mice. The ACOTSC140 cells showed tumor formation in 3/3 NOD/SCID mice while ACOTSC120 and ACOTSC132 showed tumor formation in 2/3 mice (Fig. 2A) (Suppl. Table 3). The tumor generation was faster in ACOTSC140 which showed generation of tumors in 25 days as compared to that in ACOTSC120 and ACOTSC132 that took 65 days and 140 days, respectively (Fig. 2B). The tumors generated by ACOTSC120 and ACOTSC132 were moderately differentiated squamous cell carcinoma (MDSCC) while ACOTSC140 showed well-differentiated squamous cell carcinoma (WDSCC) to MDSCC, hyperkeratinized tumors (Fig. 2C) (Suppl. Table 3).

### In vitro characterization of the cell lines

**Fig. 3:**
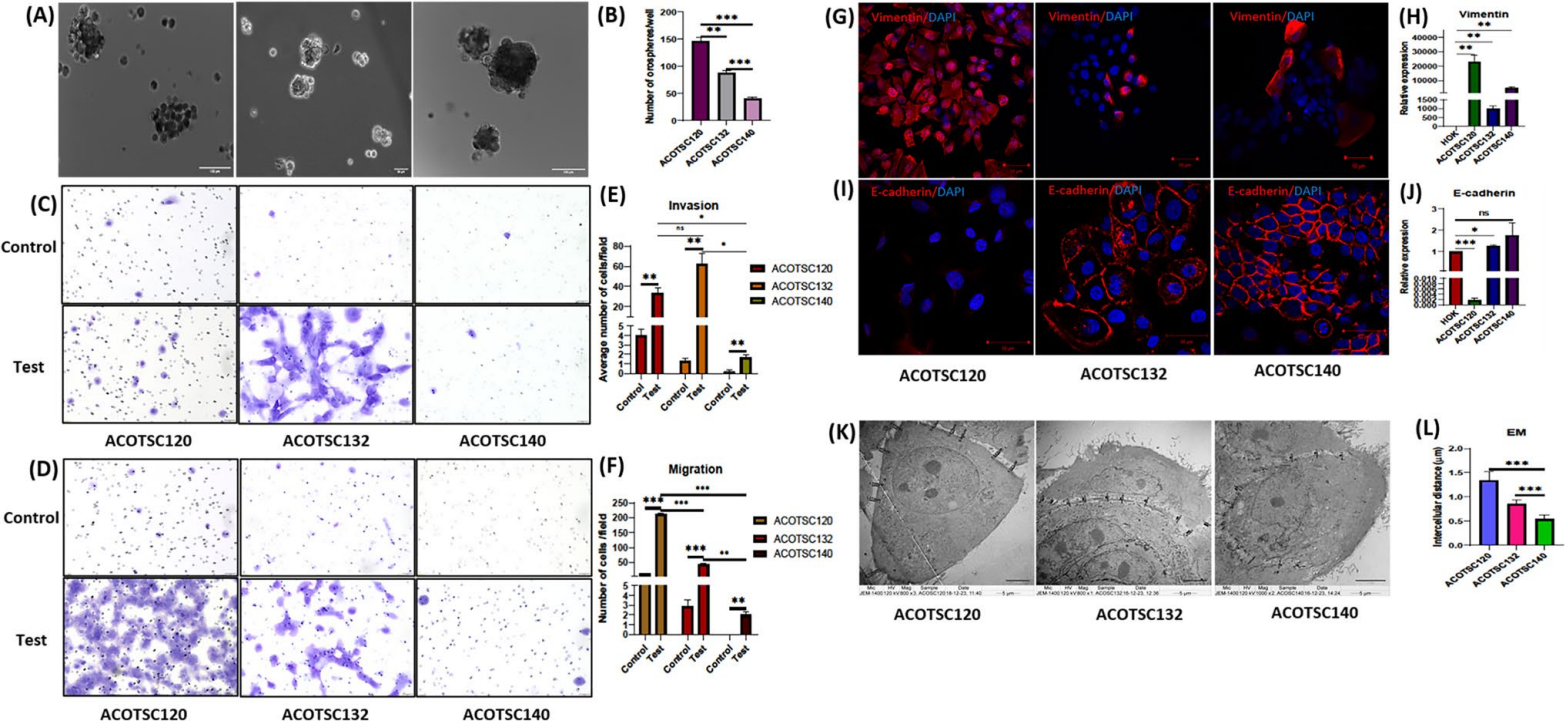
**A** The in vitro orosphere assay of ACOTSC120 (P12), ACOTSC132 (P13) and ACOTSC140 (P14) carried out in n = 3 wells for each cell line and **B** number of orospheres/well. Representative images of **C** invasion assay and **D** migration assay for ACOTSC120 (P9), ACOTSC132 (10) and ACOTSC140 (P11) carried out in *n* = 3 replicates. Graphical representation of average number of cells/field in **E** invasion assay and **F** migration assay. The IF staining of the cell lines for **G** Vimentin and **I** E-cadherin. The IF staining was carried out in three independent replicates for all three cell lines (P9, P10 and P11). The QRT-PCR assessment of **H** Vimentin and **J** E-cadherin for the cell lines. The assessment was performed on three independent replicates for all three cell lines (P9, P10 and P11) **K** Representative images electron microscopy to assess intercellular distances for ACOTSC120, ACOTSC132 and ACOTSC140. The imaging was performed on three technical replicates for all three cell lines at P20. **L** Graphical representation of intercellular distances in all three cell lines. Student’s t test was employed for statistical analysis of the data and p values are represented as ^∗^:*p* < 0.05, ^∗∗^:*p* < 0.01, ^∗∗∗^:*p* < 0.001

All three cell lines showed formation of spheroids under low-attachment, serum-starved conditions (Fig. 3A). The number of spheres was significantly higher in ACOTSC120 followed by ACOTSC132, while the ACOTSC140 cell line showed least number of spheroids per well (Fig. 3B).

To investigate the invasiveness and migratory properties, invasion and migration assays were performed which showed that ACOTSC140 was significantly least invasive and showed least migratory properties. However, ACOTSC132 cell line showed significantly the highest invasiveness, whereas the ACOTSC120 cell line showed the highest migration among the three cell lines (Fig. 3C-F).

### Expression of EMT markers and intercellular spaces between cells

To study the EMT markers in the cell lines, expression of E-cadherin and vimentin was assessed. Vimentin is expressed in early embryonic precursor cells, fibroblasts, endothelial cells and smooth muscle cells postnatally [25]. To assess the levels of E-cadherin and vimentin expression, IF staining was performed on all the three cell lines (Fig. 3G and I) and IHC was performed on the corresponding patient tumor samples **(**Suppl. Figure 1B). Vimentin expression was seen to be uniform in ACOTSC120 (Fig. 3G) with highest mRNA levels (Fig. 3H) while ACOTSC140 and ACOTSC132 showed uneven distributions (Fig. 3G) of vimentin and lower mRNA levels as compared to human oral keratinocytes (HOK) (Fig. 3H). E-cadherin is expressed in epithelial cells and involved in cell–cell adherens junctions [26]. E-cadherin was expressed uniformly in ACOTSC132 and ACOTSC140 while ACOTSC120 showed very low expression (Fig. 3I). The mRNA levels followed the same trend (Fig. 3J).

Intercellular spaces between cells were assessed using electron microscopy. The electron microscopy assessment (Fig. 3K) showed that the ACOTSC120 cells have higher intercellular spaces (average 1.34 µm) between two cells **(**Fig. 3L**)**. The ACOTSC140 had least intercellular spaces (average 0.54 µm) (Fig. 3L) while ACOTSC132 showed the intermediate distance of (average 0.86 µm) (Fig. 3L).

**Fig. 4:**
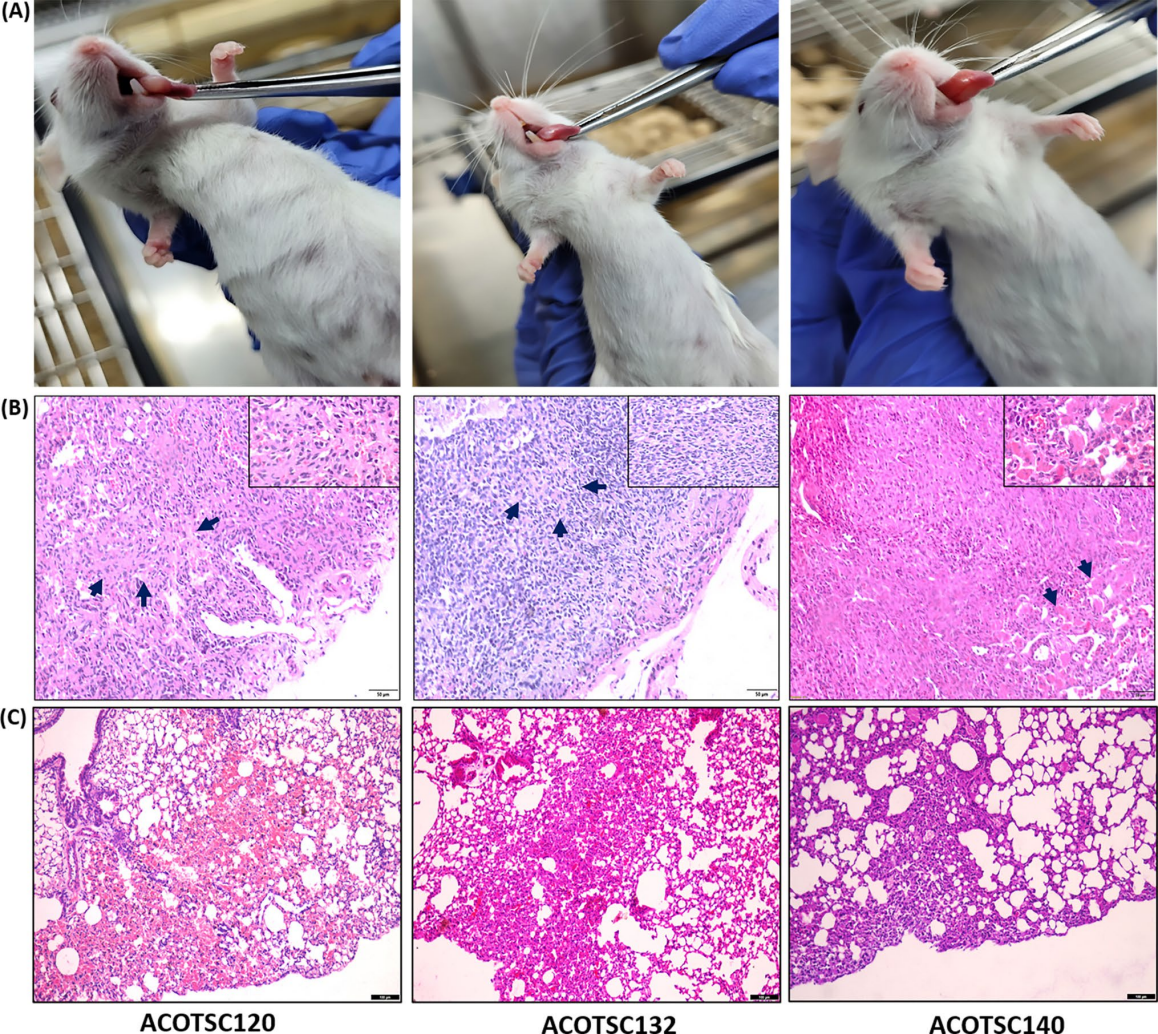
**A** Orthotopic tumor formation of ACOTSC120 (P30), ACOTSC132 (P30) and ACOTSC140 (P32) injected in mice (*n* = 3 independent biological replicates for each cell line). **B** The regional lymph nodes showing tiny metastatic foci (shown by blue arrows) **C** The H&E staining of lungs

In order to assess the in vivo metastatic potential, orthotopic tumor models of all three cell lines were generated (Fig. 4A). Peripheral cervical lymph nodes and lungs were examined by pathologist and it was observed that ACOTSC120, ACOTSC132 and ACOTSC140 showed very tiny foci of lymph node metastasis consisting of few cells (Fig. 4B), but no lung metastasis in orthotopic in vivo tongue carcinoma model (Fig. 4C).

### Evaluation of CD44 and ALDH expression

**Fig. 5:**
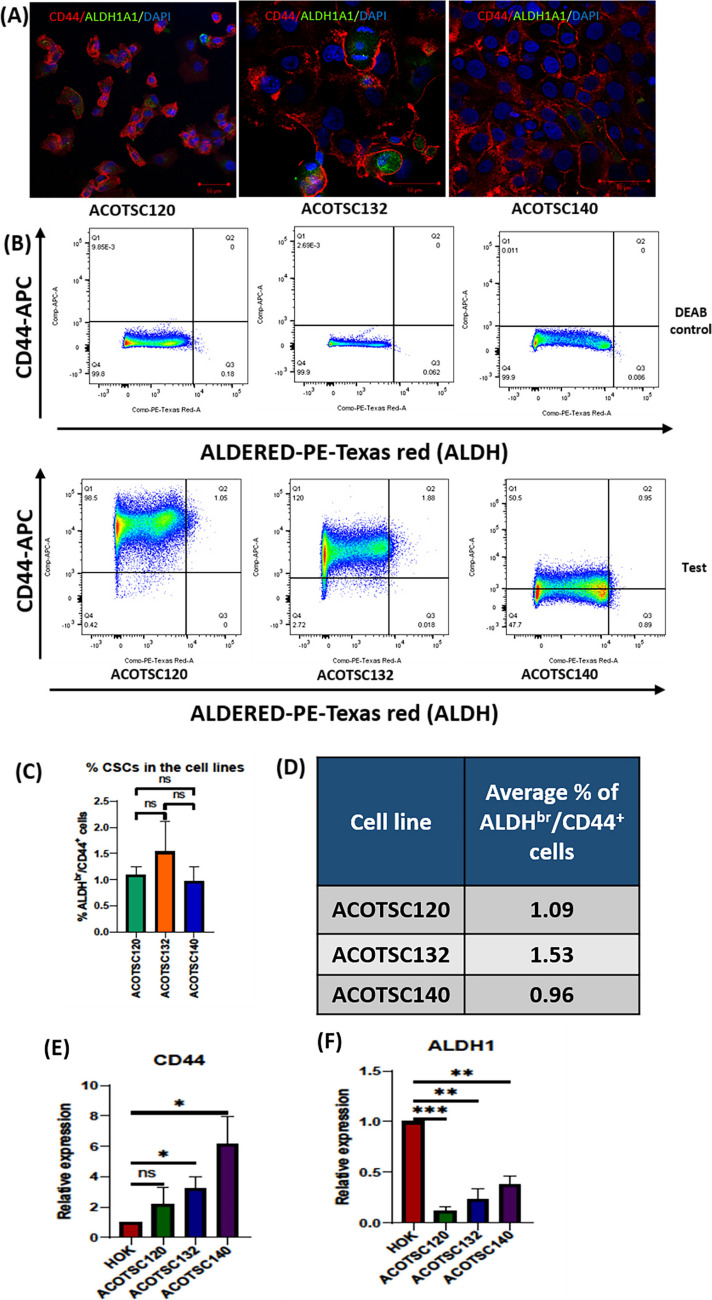
IF staining of ACOTSC120, ACOTSC132 and ACOTSC140 for **A** CD44 and ALDH1A1. The staining was performed on three independent replicates for all three cell lines (P4, P5, P6) **B** FACS analysis of ALDH^br^/CD44^+^ cells in ACOTSC120 (P21), ACOTSC132 (P22) and ACOTSC140 (P23). The FACS was performed in *n* = 3 technical replicates **C** Graphical representation of % ALDH^br^/CD44^+^ cells and **D** Average percentage of ALDH^br^/CD44^+^ cells in all three cell lines. QRT-PCR assessment of **E** CD44 and **F** ALDH1 for ACOTSC120, ACOSTC132 and ACOTSC140. The assessment was performed on three individual replicates (P9, P10, P11) for all three cell lines**.** Student’s t test was employed for statistical analysis of the data and *p* values are represented as ^∗^:*p* < 0.05, ^∗∗^:*p* < 0.01, ^∗∗∗^:*p* < 0.001

It has been reported that cells expressing CD44 [27] and aldehyde dehydrogenase (ALDH) activity [28] in head and neck squamous cell carcinoma (HNSCC) exhibit cancer stem cell (CSC)-like properties. IF staining showed that ACOTSC132 and ACOTSC120 cells showed dual positive cells, whereas ACOTSC140 showed only CD44^+^ cells (Fig. 5A). To assess the expression of ALDH^Br^/CD44^+^ cells in the cell lines, FACS analysis was done (Fig. 5B). Among the three cell lines, ACOTSC132 showed the highest average percentage of CSCs (1.53%) followed by ACOTSC120 (1.09%) and then ACOTSC140 (0.96%) (Fig. 5C,D). The CD44 expression was significantly increased in ACOTSC132 and ACOTSC140 as compared to HOK (Fig. 5E) while ALDH1 expression was decreased (Fig. 5F).

## Discussion

India bears one-third burden of global OSCC cases while among OSCC, OTSCC is the second most common cancer. Due to late diagnosis, the survival of OSCC patients is poor [1]. Carcinoma cell lines established from primary tumors serve as a powerful tool to understand the biology of the disease as well mechanisms involved in the poor treatment outcome. We report establishment of three cell lines from advanced stage treatment naïve OTSCC patient samples from Indian patients.

Abnormal DNA content is a hallmark of cancer [24]. Genomic instability might aid in tumorigenesis via amplification of oncogenes [29–32]. The karyotype and ploidy analysis showed that ACOTSC140 showed loss of Y chromosome which has been reported to correlate with overexpression of redox process and poor prognosis [32]. All three cell lines showed aneuploidy with DNA indices 1.81,1.82, and 1.72, respectively, and various abnormalities like deletions, additions and dicentric chromosomes thus make the cells a potential tool to study genomic instability and cancer.

In OSCC, HPV positive patients have shown to have a better prognosis as compared to HPV negative patients [33]. All the three cell lines were HPV negative and may serve as a platform to understand poor prognosis seen in HPV negative OSCC.

Invasion and migration assay showed that ACOTSC120 was the most migratory and second most invasive cell line. ACOTSC132 was the most invasive, while ACOTSC140 showed the least migration and invasion. The EM assessment of the cell lines showed that ACOTSC120 has the most intercellular distance which correlates with high vimentin and low E-cadherin expression and higher migration property of ACOTSC120 cells. Similarly, the ACOTSC140 cells showed the least intercellular spaces which correlated with the high E-cadherin/low vimentin expression and least invasion and migration properties. ACOTSC132 cell line seems to show intermediate EMT properties, invasion migration properties and intercellular distances. A correlation between the intercellular spaces, expression of EMT markers and the invasion and migration properties was reported in patient-derived buccal mucosa carcinoma cell lines [21] which is similar to observed in our cell lines. Additionally, the cell lines also showed very small areas of LN metastasis in in vivo orthotopic model.

All three cell lines produced spheroids in low-attachment, serum-free conditions. It has been reported in lung adenocarcinoma that spheroids show overexpression of mesenchymal markers like vimentin and downregulation of epithelial markers like E-cadherin [34]. Similarly, in our cell lines, the expression of E-cadherin correlated with the spheroid forming abilities.

Overexpression of CD44 and high ALDH activity are associated with stem-like characteristics in HNSCC [27, 28]. Therefore, expression of CD44 and ALDH was checked using IF and FACS. The highest percentage of dual positive (ALDH^br^/CD44^+^) cells was seen in ACOTSC132 followed by ACOTSC120 and ACOTSC140. The cell lines can thus be employed in studying CSCs in OTSCC.

The in vivo tumorigenic potential of the cell lines was assessed by in vivo tumorigenesis assay. To utilize a cell line to study cancer, it is crucial that the cell line is tumorigenic in vivo which is demonstrated by all three cell lines. The tumors generated by ACOTSC140 were superficial and hyperkeratinized, which correlated with the low invasiveness and migration observed. Keratin content has shown to correlate with tumor grade, metastatic property and patient prognosis in OSCC [35, 36]. Furthermore, ACOTSC120, which showed no keratinization in in vivo tumors, showed higher migration and vimentin expression while ACOTSC132 showed 30% keratinization in ½ in vivo tumors which correlated with the EMT marker expression and invasion migration properties. Therefore, there might be a correlation between tumor keratinization and EMT marker expression in tumorigenic cell line. The Indian tongue carcinoma patient cell line reported previously was seen to be non-tumorigenic [17, 18] which increases the utility of these cell lines in cancer research. Additionally, the cell lines have been established from Indian tongue cancer patients, increasing their importance in oral cancer studies in Indian patients where oral cancer is prevalent.

## Conclusion

The cell lines established from primary tumors from Indian patients with tobacco-chewing habit is a powerful tool to understand properties of the OTSCC and potentially might lead to unraveling newer targets that might lead to better treatment outcomes. The in vitro and in vivo tumorigenic property of the cell lines would give better understanding of cell behavior and enable studying properties, such as therapy resistance, metastasis, etc. The unique and varied properties of these cell lines make them potent models to study EMT, CSCs and factors affecting disease prognosis. Therefore, these cell lines are a useful tool that may enhance the understanding about properties of OTSCC.

## Supplementary Information

Below is the link to the electronic supplementary material.

Supplementary file1 (DOCX 1720 KB)

## Data Availability

The datasets generated and/or analyzed during the current study are available from the corresponding author on reasonable request.
