# Supplementary material for: Establishment and characterization of patient-derived tongue squamous cell carcinoma cell lines
Source: Hum Cell. 2025 May 20;38(4):102. doi: 10.1007/s13577-025-01231-w (PMC12092557; doi:10.1007/s13577-025-01231-w)
Supplement: Supplementary file 1 — Supplementary file1 (DOCX 1720 KB) [file 13577_2025_1231_MOESM1_ESM.docx]

**Supplementary Figures & Tables:**


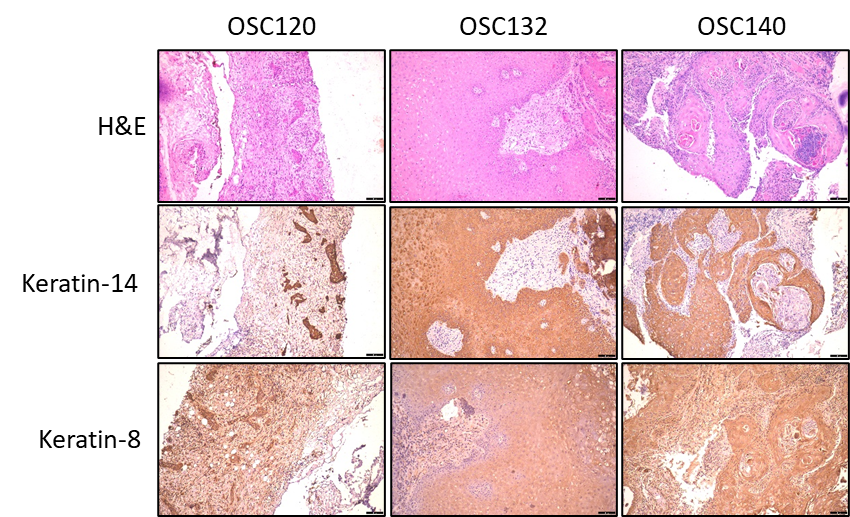


(A)


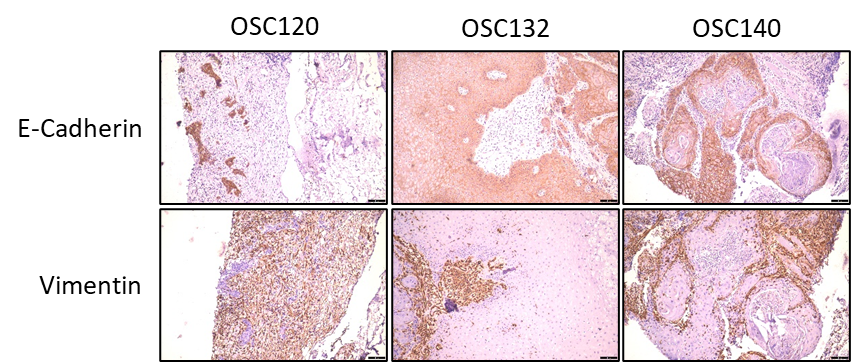


(B)

**Figure S1:** (A) The H&E staining of patient tumours and the IHC staining for Keratin-14 and Keratin-8 (B) IHC staining of E-Cadherin and Vimentin


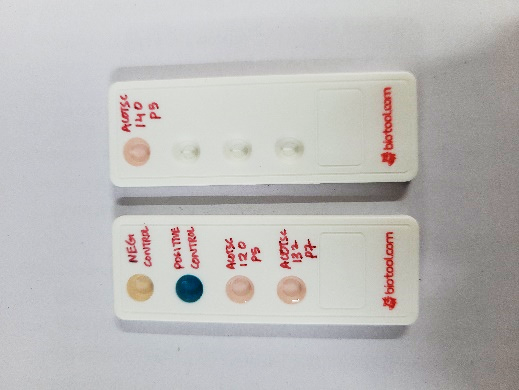


**Figure S2:** Mycoplasma detection for ACOTSC120 P5, ACOTSC132 P7 and ACOTSC140 P5 by kit method.

| **Cell line** | **Age of the patient (years)** | **Sex** | **Pathological staging** | **Grade** | **Habit** |
| --- | --- | --- | --- | --- | --- |
| ACOTSC120 | 42 | Male | T4aN3bM0 | MDSCC | Tobacco |
| ACOTSC132 | 59 | Female | T3N3bM0 | MDSCC | Mishri |
| ACOTSC140 | 37 | Male | T4aN3bM0 | MDSCC | Tobacco |

**Table S1:** Clinico-pathological features of the cell lines. MDSCC- moderately differentiated squamous cell carcinoma

STR profile of Cell lines:

| **Markers** | **ACOTSC120** | | **ACOTSC132** | | **ACOTSC140** | |
| --- | --- | --- | --- | --- | --- | --- |
| TH01 | 7 | 9 | 7 | 9 | 6 | 8 |
| D5S818 | 10 | 12 | 11 | 12 | 12 |  |
| D13S317 | 11 | 13 | 9 | 11 | 11 | 13 |
| D7S820 | 9 | 10 | 10 | 11 | 9 | 10 |
| D16S539 | 8 | 12 | 11 | 12 | 9 | 11 |
| CSF1PO | 10 | 12 | 11 |  | 11 | 12 |
| vWA | 18 |  | 14 | 18 | 14 | 16 |
| TPOX | 10 |  | 11 |  | 8 | 11 |
| Amelogein | X | Y | X |  | X |  |

STR profile of patient samples:

| **Markers** | **OSC120** | | **OSC132** | | **OSC140** | |
| --- | --- | --- | --- | --- | --- | --- |
| TH01 | 7 | 9 | 7 | 9 | 6 | 8 |
| D5S818 | 10 | 12 | 11 | 12 |  | 12 |
| D13S317 | 11 | 13 | 9 | 11 | 11 | 13 |
| D7S820 | 9 | 10 | 10 | 11 | 9 | 10 |
| D16S539 | 8 | 12 | 11 | 12 | 9 | 11 |
| CSF1PO | 10 | 12 | 11 |  | 11 | 12 |
| vWA |  | 18 | 14 | 18 | 14 | 16 |
| TPOX | 10 |  | 11 |  | 8 | 11 |
| Amelogein | X | Y | X | X | X | Y |

**Table S2:** STR profile of the cell lines and the corresponding patient sample

| Cell line | Mice | % tumour content | Grade |
| --- | --- | --- | --- |
| ACOTSC120 | 1 | 85 | Moderately differentiated squamous cell carcinoma (MDSCC) |
|  | 2 | 85 | MDSCC |
|  | 3 | No tumour | - |
| ACOTSC132 | 1 | 30 | MDSCC With 30% keratin |
|  | 2 | 90 | MDSCC |
|  | 3 | No tumour | - |
| ACOTSC140 | 1 | 35 | Well differentiated squamous cell carcinoma (WDSCC) to MDSCC, Hyperkeratinized, Superficial |
|  | 2 | 40 | MDSCC, hyperkeratinized |
|  | 3 | 65 | WDSCC to MDSCC, hyperkeratinized |

**Table S3:** Tumour content of tumours derived through *in-vivo* tumorigenesis

| **Primer** | **Oligonucleotide sequence in 5’ to 3’ orientation** |
| --- | --- |
| GAPDH | Forward: GAAGGTCGGAGTCAACGGATTT  Reverse: GATGACAAGCTTCCCGTTCTCA |
| Vimentin | Forward: AGTCCACTGAGTACCCGGAGAC  Reverse: CATTTCACGCATCGGCGTTC |
| E- cadherin | Forward: CGAGAGCTACACGTTCACGG  Reverse: GGGTGTCGAGGGAAAAATAGG |
| ALDH1 | Forward: CTGCTGGCGACAATGGAGT  Reverse: GTCAGCCCAACCTGCACAG |
| CD44 | Forward: TCCAACACCTCCCAGTATGACA  Reverse: GGCAGGTCTGTGACTGATGTACA; |
| MY09 | 5'‑CGTCCMARRGGAWACTGATC‑3' |
| MY11 | 5'‑GCMCAGGGWCATAAYAATGG‑3' |
| GP5+ | 5'-TTTGTTACTGTGGTAGATACTAC-3' |
| GP6+ | 5'-GAAAAATAAACTGTAAATCATATTC-3' |

**Table S4:** List of Real-time primers

| **Antigen/Antibody** | **Dilution /Application** | **Source** |
| --- | --- | --- |
| **Fluorescence activated cell sorting (FACS)** | | |
| CD44 APC |  | BD Pharmingen |
| **Immunofluorescence Assay (IFA)** | | |
| Keratin-14 | 1:100 | Abcam |
| Keratin-8 | 1:100 | Novus biologicals |
| E-cadherin | 1:100 | Abcam |
| Vimentin | 1:100 | Abcam |
| CD44 | 1:600 | Cell signalling technologies |
| ALDH1A1 | 1:100 | Abcam |
| Anti-mouse conjugated with Cyanin-3 (Cy3) | 1:200 | Jackson immunoresearch |
| Anti-rabbit conjugated with Alexa Fluor 568 (A568) | 1:200 | Abcam |
| Anti-rabbit conjugated with fluorescein isothiocyanate (FITC) | 1:200 | Jackson immunoresearch |

**Table S5:** List of the primary and secondary antibodies
